# Yes‐associated protein is essential for proliferative vitreoretinopathy development via the epithelial‐mesenchymal transition in retinal pigment epithelial fibrosis

**DOI:** 10.1111/jcmm.16958

**Published:** 2021-10-01

**Authors:** Wei Zhang, Jing Li

**Affiliations:** ^1^ Tianjin Key Lab of Ophthalmology and Visual Science Tianjin Eye Institute Tianjin Eye Hospital Clinical College of Ophthalmology Tianjin Medical University Tianjin China; ^2^ Department of Ophthalmology Tianjin Medical University General Hospital Tianjin China

**Keywords:** epithelial‐mesenchymal transition, proliferative vitreoretinopathy, retinal pigment epithelial cells, transforming growth factor‐β, yes‐associated protein

## Abstract

This study was aim to investigate whether the progression of proliferative vitreoretinopathy (PVR) depended on the activation of Yes‐associated protein (YAP) and the subsequent epithelial‐mesenchymal transition (EMT) of retinal pigment epithelial (RPE) cell. The effect of YAP activation on retinal fibrosis in a PVR mouse model and in human ARPE‐19 cells in vitro was studied. After treated with transforming growth factor‐β2(TGF‐β2), the expressions of fibrogenic molecules, YAP activation and the TGF‐β2‐Smad signalling pathway in ARPE‐19 cells were detected by Western blot and immunocytochemical analyses. The effect of YAP on change in fibrosis and EMT was tested by knockdown experiment using verteporfin (YAP inhibitor). YAP was upregulated in the PVR mouse model and during TGF‐β2–induced RPE cell EMT. In an in vivo study, verteporfin attenuated PVR progression in a mouse model. Additionally, YAP knockdown retained phenotype of RPE cells and ameliorated TGF‐β2–induced migration, gel contraction and EMT in vitro. YAP knockdown inhibited the TGF‐β2–induced upregulation of connective tissue growth factor (CTGF), smooth muscle actin (SMA‐α) and fibronectin. YAP was essential for the TGF‐β2–induced nuclear translocation and phosphorylation of Smad2/3. Our work provides direct evidence that YAP is an essential regulator of EMT and profibrotic responses in PVR and indicates that YAP inhibition could be a potential target in PVR therapeutic intervention.

## 1 INTRODUCTION

Proliferative vitreoretinopathy (PVR) is a tissue response that is characterized by the proliferation of many types of cells on the surface of the retina and within the vitreous gel, leading to epiretinal membrane formation and traction on the retina.^1^ In addition, PVR is a main reason of failed retinal detachment (RD) surgery, which occurs in 5%–10% of RD patients.^2^ Despite advances in surgery and adjuvant treatments, the incidence of PVR can be expected to be high even after successful primary or complicated RD surgery.^3^ Epiretinal fibrotic membranes could be found in the PVR, and epiretinal changes are also critical in most cases.^4^ Cells, involving glial cells, phagocytes, fibroblasts and retinal pigment epithelial (RPE) cells, have been observed in the retinal membranes of PVR patients and experimental animal models.^5^ The reason why certain RD cases develop PVR has not been fully elucidated.

Retinal pigment epithelial cell plays a key role in PVR progression due to RPE cell is the main cellular component within the PVR membrane.^6^ Previous researchers have observed a substantial role of epithelial‐mesenchymal transition (EMT) in RPE cells during the PVR process.^6^, ^7^ RPE cells diffuse into the vitreous cavity through holes in the detached retina, then transform into profibrotic cells and migrate both on the surface of retina and beneath the retina, ultimately resulting in the onset and progression of PVR.^8^ This process is characterized by cell migration accompanied by extracellular matrix generation, membrane formation, proliferative membrane contraction and eventually RD contraction.^9^, ^10^ Transforming growth factor‐beta (TGF‐β) is observed at much higher levels in the vitreous fluid of eyes from PVR patients than in uncomplicated eyes from RD patients, and TGF‐β is also the main driving force that leads to EMT in many cells, especially in RPE cells.^11^ However, inhibition of TGF‐β activity alone does not contribute to preventing the incidence of PVR in RD patients. Therefore, it is necessary to better understand the exact mechanism of PVR development and discover novel therapeutic targets for PVR.

The Hippo signalling pathway is a highly conserved threonine/serine kinase cascade. This pathway was found to play a key role in the process of fibrosis via the effects of two core proteins, namely WW domain‐containing transcription regulator 1 (also referred to herein as TAZ) and yes‐associated protein (YAP).^12^ At the molecular level, the expression of connective tissue growth factor (CTGF) is modulated by the YAP and TAZ protein (YAP/TAZ) complex.^13^ In addition, substrate stiffness alters the cytoplasmic and nuclear localization of the YAP protein, leading to the hypothesis that the YAP protein may act to mediate mechanical conduction through an unknown pathway.^14^ It was also found that the expression of CTGF is closely associated with the localization of YAP protein in endothelial cells and mesenchymal stem cells.^15^ Because CTGF regulates a variety of cellular processes, CTGF is significantly upregulated in several retinal gliosis conditions, such as age‐related macular degeneration, PVR and proliferative diabetic retinopathy.^16^, ^17^ From a clinical and translational perspective, exploring the mechanical effects of YAP activation may help develop new therapies for PVR.

Previously, we demonstrated that YAP is a key regulator of profibrotic response in hyperglycaemia‐induced retinal fibrosis.^18^ We also found that YAP activation enhances both the proliferation and fibrogenic activity of Müller cells via the PI3K/Akt pathway.^19^ Based on these observations, we used experimental PVR models to investigate how PVR development depends on YAP activation and the subsequent EMT in RPE cells. Our results provide direct evidence that YAP is an essential regulator of EMT and profibrotic responses in PVR and will help identify effective treatment for preventing the onset of PVR.

## 2 MATERIALS AND METHODS

### 2.1 Proliferative vitreoretinopathy (PVR) induction and treatment

The study was approved by the medical ethics committee of Tianjin Medical University and was conducted in comply with the Declaration of Helsinki. Our experiment was performed in comply with the approved guideline. The C57BL/6 mouse (6–8 weeks old) model of PVR was established by intravitreal injection of dispase as previously described.^20^ In short, intravitreal injection was performed in the dorsonasal quadrant 1.5 mm from the limbus of the right eye. A 30‐G Hamilton syringe was used to intravitreally inject 3 µl of Dispase II (Sigma‐Aldrich, concentration of 0.2 U/µl). The control mice received intravitreal injections of 3 µl of sterile saline solution. To inhibit the interaction of YAP‐TEAD in vivo, we intraperitoneally administered verteporfin (100 mg/kg every other day for 8 weeks) after establishment of the PVR mouse model. After the mice were euthanized, the eyes were quickly removed and processed for immunohistochemical and Western blot analyses as described in the following sections.

### 2.2 ARPE‐19 cells culture and treatment

Human retinal pigment epithelial cell line (ARPE‐19) was purchased from American type culture collection (ATCC) and cultured in Dulbecco's modified Eagle's medium (DMEM)/F12 medium, which contained 1% foetal bovine serum at 37°C with 5% CO_2_. The ARPE‐19 cells were used at passages 2–4 in all the experiments. To inhibit the expression of YAP in ARPE‐19 cells, ARPE‐19 cells were treated with siRNA against YAP. Nonsilencing siRNA and YAP‐siRNA was purchased from Thermo Fisher Scientific. Transient transfection of siRNA was performed using Lipofectamine transfection reagent 2000. To inhibit the interaction of YAP‐TEAD in vitro, ARPE‐19 cell was treated with verteporfin at a concentration of 100 ng/ml for 48 h as recommended by the manufacturer. Recombinant TGF‐β2 (Santa Cruz Biotechnology) was used to induce EMT in ARPE‐19 cells at a concentration of 10 ng/ml. A constitutively active YAP mutant (YAP5SA) was purchased from Invitrogen. YAP5SA has its LATS kinase‐induced phosphorylation sites substituted for alanine to prevent cytoplasmic sequestration and proteasomal degradation.^21^

### 2.3 Collagen gel contraction

A collagen gel contraction assay was performed as previously described by Yao et al.^22^ Before adding the cells, 24‐well plates were coated with 1 ml of 1% BSA for 1 h at 37°C. The ARPE‐19 cells were collected and mixed with type I collagen at a concentration of 2 mg/ml (Invitrogen) after treatment with TGF‐β2 with or without verteporfin pretreated. This mixture was then added to a 24‐well plate precoated with BSA and incubated in 5% CO_2_ for 1 h at 37°C to catalyse gel polymerization. After 48 h, took a picture and quantified the ratio of contraction area of the gel.

### 2.4 Analysis of ARPE‐19 cell proliferation, migration and invasion

The proliferation of ARPE‐19 cells was assessed using a bromodeoxyuridine (BrdU)‐ELISA (Santa Cruz Biotechnology). In short, ARPE‐19 cells were treated with 10 ng/ml TGF‐β2 in DMEM/F12 for 24 h and incubated with BrdU for 2 h. The optical density was determined with a microplate reader. The proliferative activity of ARPE‐19 cells was represented by the optical density (OD) of each wells minus that of the blank wells.

The migration of ARPE‐19 cells was evaluated with an Oris 96‐well cell migration assay kit (Santa Cruz Biotechnology). In short, ARPE‐19 cells were seeded in each well and treated with DMEM/F‐12 containing 10 ng/ml TGF‐β2 for 1 h. Then, the cells were treated with 5 mM aphidicolin to inhibit cell division, cultured for 48 h and stained with calcein AM in PBS for another 1 h. A Spectra microplate reader was used to measure the signal intensity of the stained ARPE‐19 cells that migrated into the detection area.

The invasion of ARPE‐19 cells was measured by Transwell assay. Briefly, cells were seeded into the upper wells, 10 ng/ml TGF‐β2 in DMEM/F‐12 was applied to the lower wells, and then, the plates were incubated for 24 h. The ARPE‐19 cells remaining in the upper well were removed by wiping. The ARPE‐19 cells that migrated onto the lower side of the filter were fixed and stained with 100% methanol. An optical microscope was used to observing the stained cells and measuring the migratory activity of ARPE‐19 cells.

### 2.5 Immunocytochemistry

Immunocytochemistry was performed according to our previous research.^18^ In short, ARPE‐19 cells were treated with 4% paraformaldehyde and then blocked with 10% foetal bovine serum. Next, incubated the cells with the antibodies overnight against fibronectin, SMA‐α, CTGF and Smad2/3, which were purchased from Abcam Biotechnology. Then, the ARPE‐19 cell was probed with goat anti‐mouse or anti‐rabbit IgG secondary antibody (Abcam Biotechnology) and fixed with an anti‐fading reagent containing DAPI. The fluorescence microscope was used to obtain images.

### 2.6 Histological and immunohistochemical measurements

Masson's trichrome staining and immunohistochemical analysis were performed according to our previous research.^18^ In short, prepared eye section from the paraffin embedded tissue and treated the slide with solution of phosphomolybdic acid as a mordant for 10 min and then immersed in methyl‐blue. Thereafter, washed the slide in PBS and then treated with 1% solution of acetic acid for 2 min. Incubated the slide with antibody against YAP (Abcam Biotechnology) and then stained the slide with biotinylated anti‐mouse IgG secondary antibody (Abcam Biotechnology) for 2 h. Next, incubated the slide with horseradish peroxidase‐conjugated streptavidin for 1 h. The fluorescence microscope was used to obtain images.

### 2.7 Real‐time PCR

Total RNA was isolated from ARPE‐19 cells by the TRIzol reagent. SuperScript first‐strand synthesis system was used to reverse transcribed into cDNA. Then performed quantitative PCR using the Power SYBRGreen PCR MasterMix. The primer sequences were shown as the YAP primers were as follows: Fwd 5′‐CTGGAGGGAGATGGAATGAA‐3′; Rev 5′‐ATCGCCTTA GCTCCTTCACA‐3′; the β‐actin primers were: Fwd5′‐CCTCTATGCCAACACAGTGC‐3′; Rev 5′‐CCTGCTTGCTGATCCACATC‐3′.

### 2.8 Western blotting analysis

Total protein was extracted from ARPE‐19 cells and retinal samples from mice. As described in a previous report,^19^ cytoplasmic and nuclear protein was gradually prepared from ARPE‐19 cells or retinal samples using cytoplasmic and nuclear extraction reagent kits (Santa Cruz Biotechnology). The antibodies against Collagen I, SMA‐α, CTGF, fibronectin, YAP, TGF‐β1, E‐cadherin, ZO‐1, Smad2/3 and β‐actin were obtained from Santa Cruz Biotechnology. An enhanced chemiluminescence kit (Abcam Biotechnology) was used to detect the target protein. Imaging the blot using Olympus soft image solutions GmbH.

### 2.9 Statistical analysis

The data were expressed as the mean ± SEM, and the data were compared using one‐way analysis of variance (ANOVA). *p* value < 0.05 was considered statistically significant. The Mann‐Whitney statistical test was used for statistical analysis for comparison involving two groups of data. All the data were analysed by using a statistical software package (SPSS 18.0).

## 3 RESULTS

### 3.1 YAP is activated during the development of proliferative vitreoretinopathy

**FIGURE 1:**
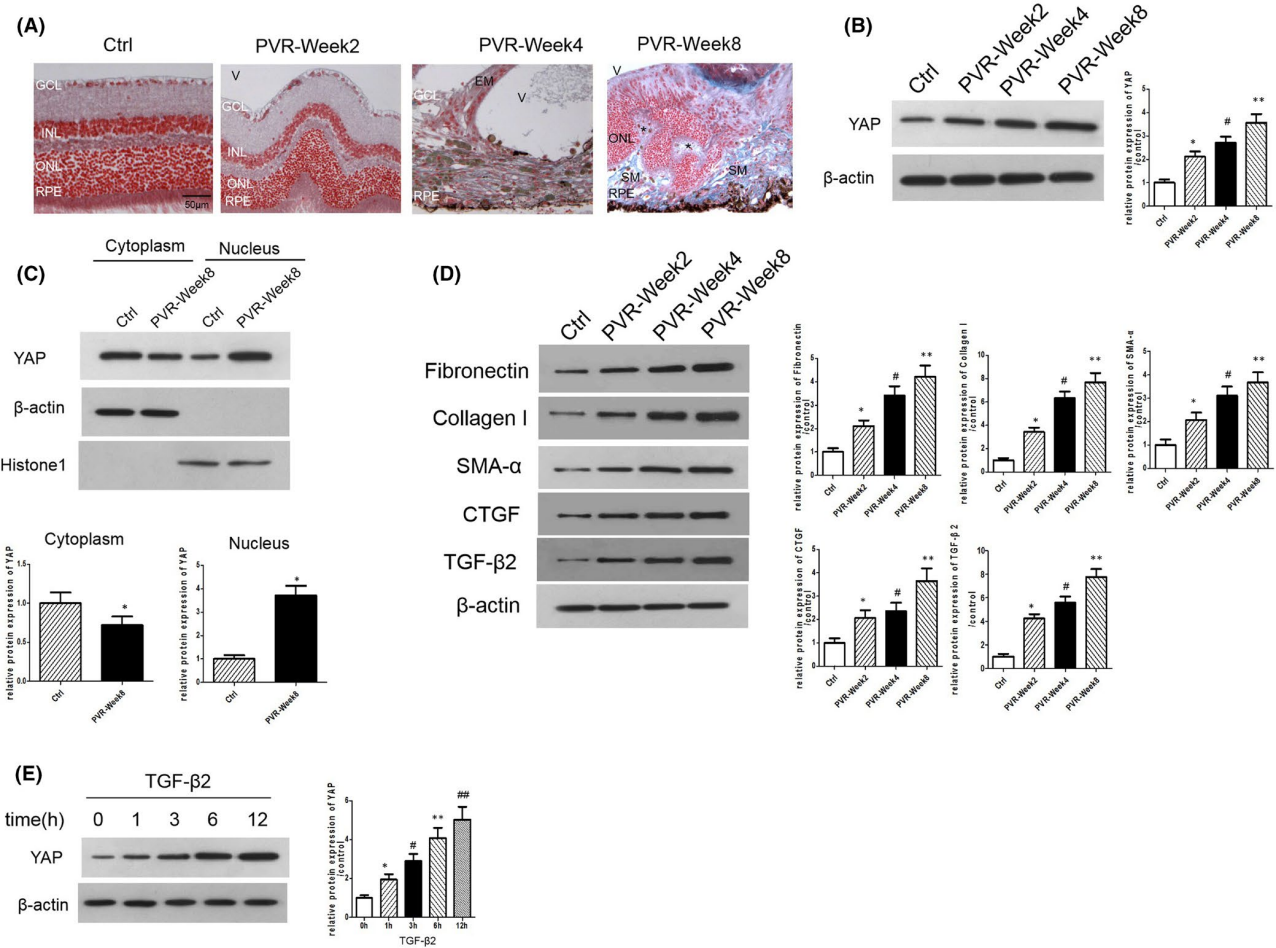
YAP is activated during the development of proliferative vitreoretinopathy. (A) Representative Masson staining in the retinas from different groups. In PVR‐Week2 group, it showed the presence of retinal fold; in PVR‐Week4 group, it showed a fibrous epiretinal membrane (EM) attached to the inner retinal layer. In addition, the normal structure of retina was disrupted. In PVR‐Week8 group, it showed subretinal membrane (SM) attached to the RPE layer within the subretinal space. The structure of outer nuclear rosette was shown, and the normal structure of retina was completely disrupted. GCL, ganglion cell layer; INL, inner nuclear layer; ONL, outer nuclear layer; RPE, retinal pigment epithelial; V, vitreous; EM, epiretinal membrane; SM, subretinal membrane; *, outer nuclear rosette. Scale bar: 50 = µm. (B) Western blot assay for YAP level in the retinas of mice. *p < 0.05 versus control group; ^#^ *p* < 0.05 versus PVR‐Week2 group; **p < 0.05 versus PVR‐Week4 group; *n* = 3. (C) Expression of YAP in the cytoplasm and nucleus of the retinas of mice was measured by Western blotting. *p < 0.05 versus control group; *n* = 3. (D) Western blot assay for the protein levels of fibronectin, collagen I, SMA‐α, CTGF and TGF‐β2 in the retinas of mice. *p < 0.05 versus control group; ^#^ *p* < 0.05 versus PVR‐Week2 group; **p < 0.05 versus PVR‐Week4 group; *n* = 3. (E) YAP level in ARPE‐19 cells treated with TGF‐β2 was measured by Western blotting. *p < 0.05 versus control group; ^#^ *p* < 0.05 versus TGF‐β2(1 h) group; **p < 0.05 versus TGF‐β2(3 h) group; ^##^ *p* < 0.05 versus TGF‐β2(6h) group; *n* = 3

Masson staining showed that the presence of retinal fold in PVR‐Week2 group (Figure 1A). In PVR‐Week4 group, it showed a fibrous epiretinal membrane (EM) attached to the inner retinal layer (Figure 1A). In PVR‐Week8 group, it showed subretinal membrane (SM) attached to the RPE layer within the subretinal space (Figure 1A). The structure of outer nuclear rosette was shown, and the normal structure of retina was completely disrupted. YAP expression greatly increased as early as 2 weeks after PVR induction and reached a peak at 8 weeks (Figure 1B). Due to activated YAP performs its pro‐transcriptional function in the nucleus, we separated the cytoplasmic and nuclear proteins from the retinas and found that PVR elevated the expression of YAP and promoted the translocation of most of the YAP proteins into the nucleus (Figure 1C). Furthermore, Western blot analysis also showed increased expression of SMA‐α and CTGF (targets of YAP) as well as accumulation of ECM, collagen I and fibronectin in the retinas of mice with PVR (Figure 1D). TGF‐β2 is a strong stimulus of RPE activation, and our study revealed increased TGF‐β2 expression in the retinas of mice with PVR (Figure 1D). In order to simulate the PVR environment in Vitro, we added TGF‐β2(at the concentration of 10 ng/ml) to the medium of ARPE‐19 cells and detected YAP level in the process of EMT. Our results indicated that the protein level of YAP greatly upregulated when treated with TGF‐β2 for 48 h (Figure 1E).

### 3.2 YAP inhibition reverses the TGF‐β2–induced activation of cell motility and gel contraction

**FIGURE 2:**
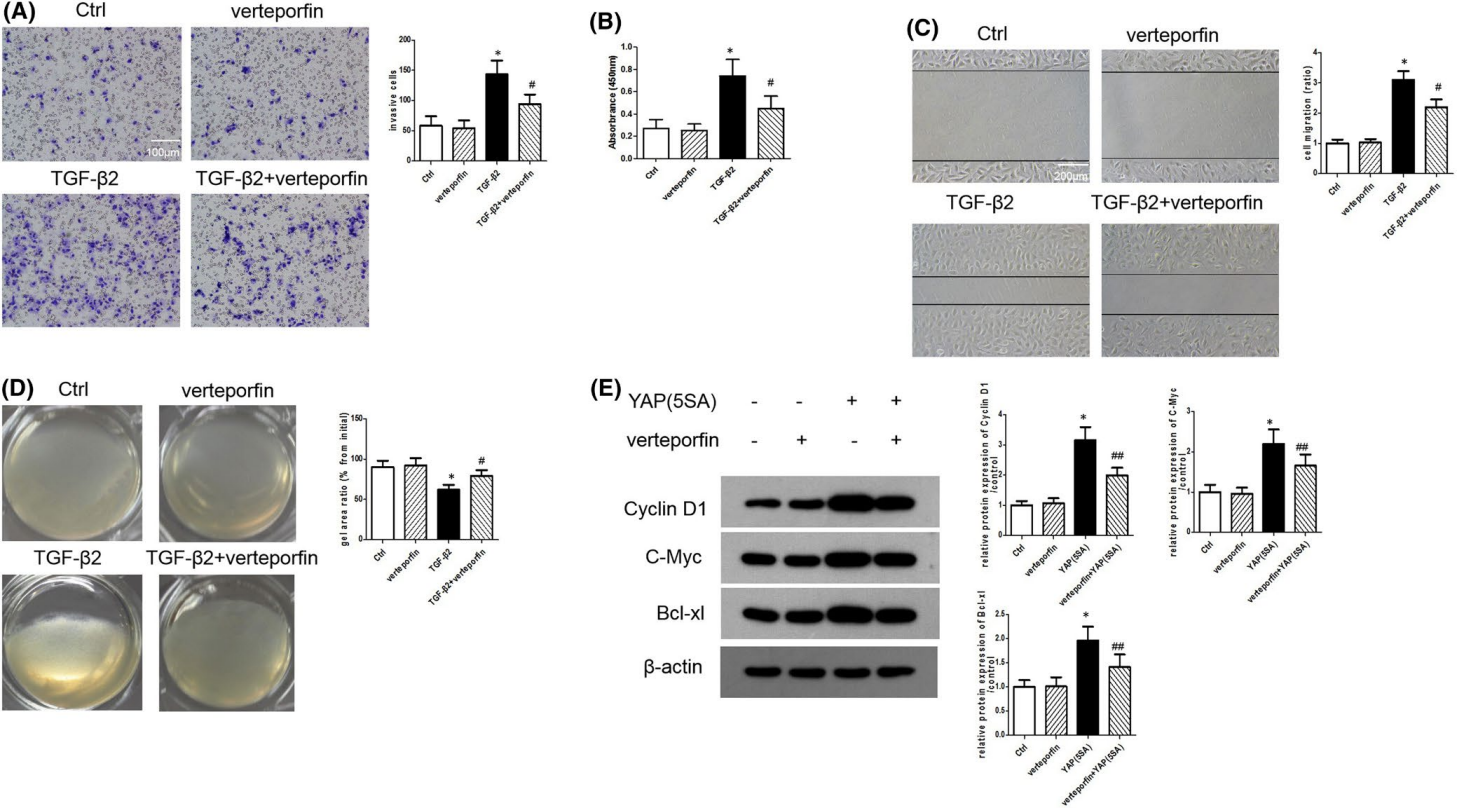
YAP Inhibition rescues TGF‐β2‐stimulated activation of cell motilities and gel contraction. (A) Invasion of ARPE‐19 cells treated with TGF‐β2 or/and verteporfin was detected by transwell assays. Scale bar = 100 µm. (B) Proliferation of ARPE‐19 cells treated with TGF‐β2 or/and verteporfin was analysed by BrdU‐ELISA assays. (C) Migration of ARPE‐19 cells treated with TGF‐β2 or/and verteporfin was analysed by Scratch assays. Scale bar = 200 µm. (D) Contractile function of ARPE‐19 cells treated with TGF‐β2 or/and verteporfin was detected by collagen gel contraction assay. (E) The protein levels of Cyclin D1, C‐Myc and Bcl‐xl in ARPE‐19 cells expressing constitutive active YAP (YAP5SA) with or without verteporfin treatment were detected by Western blotting. *p < 0.05 versus control group; ^#^ *p* < 0.05 versus TGF‐β2 group; ^##^ *p* < 0.05 versus YAP5SA group; *n* = 3

Because YAP is activated during PVR progression in vivo and in vitro, we were interested in determining whether YAP inhibition could suppress the changes in RPE cell behaviour observed during culture in a PVR environment. To further explore the role of the YAP‐TEAD complex in the TGF‐β2–stimulated activation of RPE cells, we treated ARPE‐19 cells with verteporfin, which could inhibit the interaction of YAP‐TEAD. TGF‐β2–induced invasion was markedly inhibited by verteporfin pretreatment compared with control pretreatment in the modified Boyden chamber assay (Figure 2A). The BrdU‐ELISA results showed that verteporfin prevented the TGF‐β2–induced increase in the proliferative ability of ARPE‐19 cells (Figure 2B). We also found that verteporfin significantly suppressed the TGF‐β2–induced increase in the migratory ability of ARPE‐19 cells (Figure 2C). In addition, collagen gel contraction assay showed that TGF‐β2 could induce the contraction of collagen gel containing ARPE‐19 cells, which was effectively inhibited by verteporfin treatment (Figure 2D). The activation of cyclin D‐dependent kinase is crucial for the transition from the G1 phase to the S phase, which is essential for regulating the entry and exit of cell cycle phases in the process of cell proliferation.^23^ Western blot assay showed that ARPE‐19 cells expressing constitutively active YAP (YAP5SA) increased the protein levels of Cyclin D1, C‐Myc, Bcl‐xl, even in the absence of TGF‐β2, suggesting that YAP activation is essential the proliferation of ARPE‐19 cells (Figure 2E). In addition, verteporfin treatment attenuated YAP5SA‐induced higher levels of Cyclin D1, C‐Myc and Bcl‐xl (Figure 2E). These findings suggested that YAP knockdown contributed to a decrease in the RPE cell motility and gel contraction following TGF‐β2–induced EMT changes.

### 3.3 YAP inhibition reverses TGF‐β2–induced EMT in RPE cells

**FIGURE 3:**
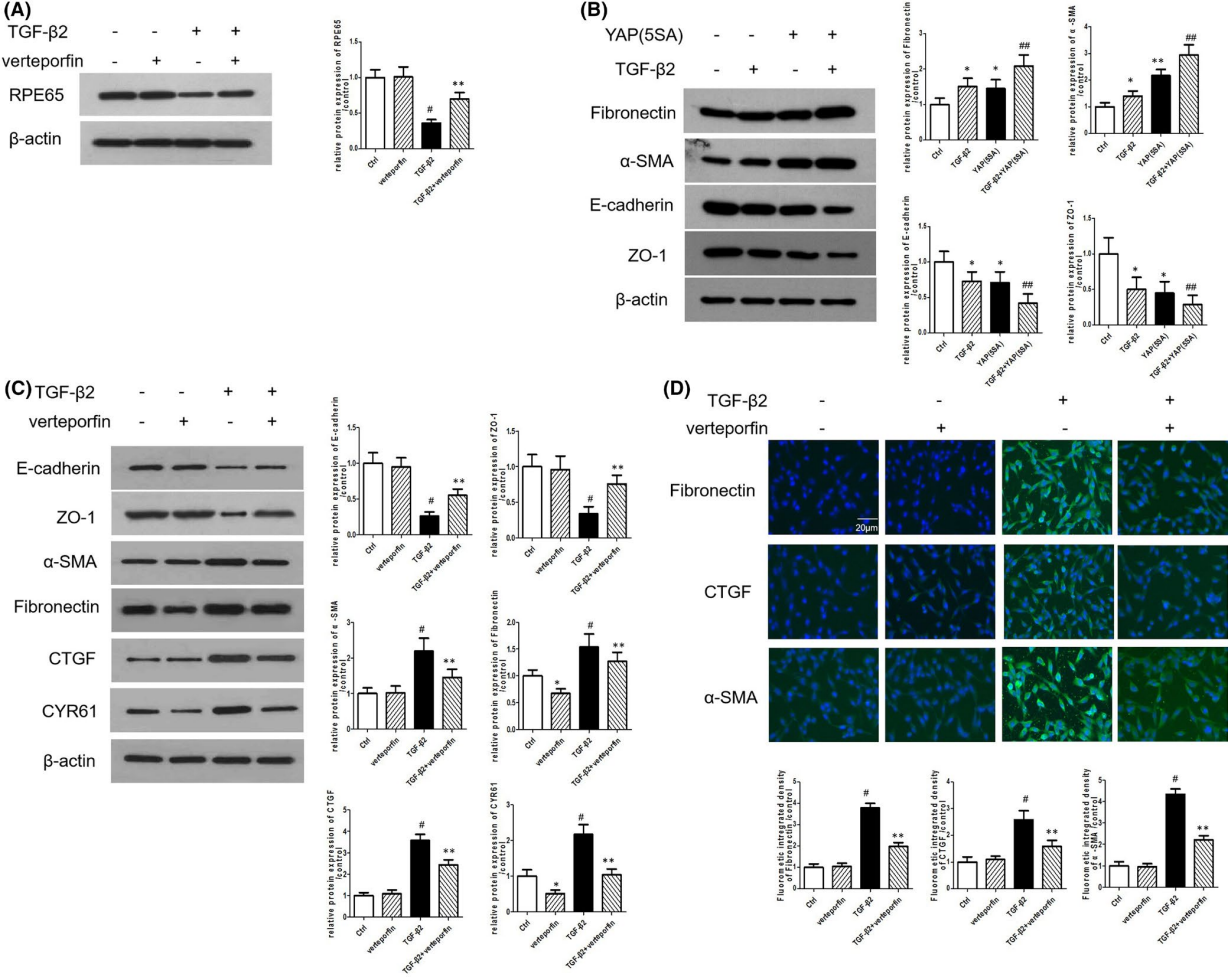
YAP Inhibition rescues TGF‐β2‐stimulated EMT in RPE cells. (A) Protein level of RPE65 in ARPE‐19 cells exposure to TGF‐β2 or/and verteporfin for 24 h were detected by Western blotting. (B) Protein levels of fibronectin, α‐SMA, E‐cadherin and ZO‐1 in ARPE‐19 cells expressing constitutive active YAP (YAP5SA) with or without TGF‐β2 were measured by Western blotting. (C) Protein levels of ZO‐1, E‐cadherin, α‐SMA, fibronectin, CTGF and CYR61 in ARPE‐19 cells exposure to TGF‐β2 or/and verteporfin for 24 h were measured by Western blotting. (D) Expression of fibronectin, CTGF and ɑ‐SMA in ARPE‐19 cells exposure to TGF‐β2 or/and verteporfin was detected by Immunofluorescence staining. Scale bar = 20 µm. *p < 0.05 versus control group; ^#^ *p* < 0.05 versus verteporfin group; **p < 0.05 versus TGF‐β2 group; ^##^ *p* < 0.05 versus YAP5SA group; *n* = 3

An important feature of RPE cells in the process of PVR was the loss of specific marker of RPE such as RPE65 protein.^24^ Our results revealed that TGF‐β2 reduced the expression of RPE65 protein, while verteporfin reversed this effect (Figure 3A). Secondly, we found that ARPE‐19 cells expressing constitutively active Yap (5SA) increased the levels of the mesenchymal marker fibronectin and α‐SMA and reduced the levels of epithelial marker E‐cadherin and ZO‐1(Figure 3B), suggesting that YAP activation is essential for the EMT of ARPE‐19 cells. In addition, TGF‐β2 treatment had the same effects as ARPE‐19 cells expressing constitutive active Yap5SA (Figure 3B). Moreover, TGF‐β2 stimulation reduced the levels of epithelial marker E‐cadherin and ZO‐1 and upregulated the levels of the mesenchymal marker fibronectin and α‐SMA in ARPE‐19 cells, whereas verteporfin attenuated these effects of TGF‐β2 at the protein level (Figure 3C). In addition, Western blot assays also showed that TGF‐β2 could stimulate the translation of the YAP downstream genes CTGF and CYR61, while verteporfin could effectively inhibit the increased CTGF and CYR61 translation induced by TGF‐β2 (Figure 3C). In addition, immunofluorescence examination showed that verteporfin prevented the TGF‐β2–induced increasing of fibronectin, CTGF and α‐SMA (Figure 3D).

### 3.4 YAP inhibition suppresses experimental PVR progression in vivo

**FIGURE 4:**
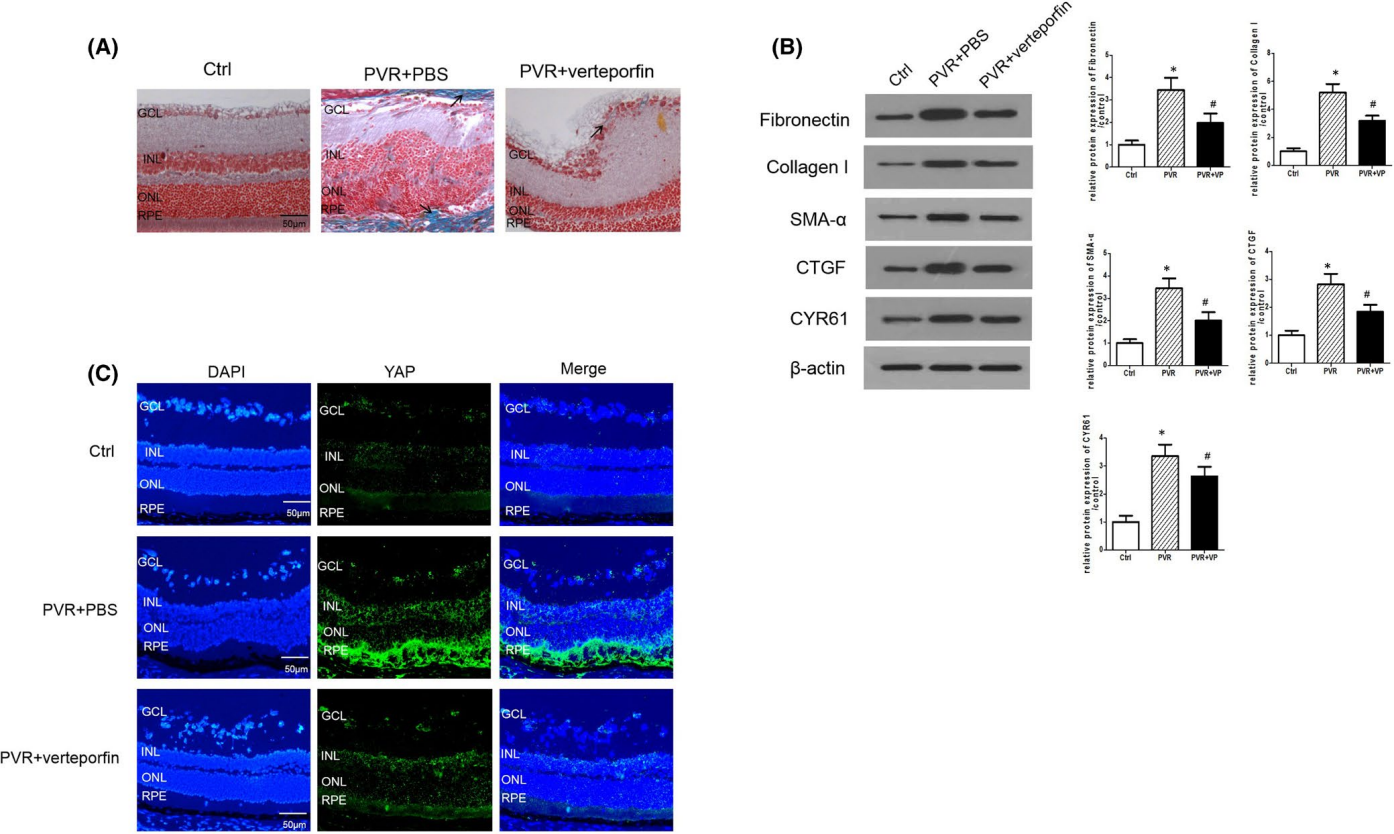
YAP Inhibition suppresses experimental PVR progression in vivo. (A) Representative Masson staining in the retinas of PVR mouse models with or without vertoporfin. (Arrow shows the collagen fibres.) (B) Western blot assay for protein levels of CTGF, CYR61, ɑ‐SMA, collagen I and fibronectin in the retinas of mouse with or without vertoporfin. (C) Immunofluorescence staining of YAP in the retinas. The quantitative assay was done using Image Pro Plus software. GCL, ganglion cell layer; INL, inner nuclear layer; ONL, outer nuclear layer; RPE, retinal pigment epithelial. Scale bar: 50 = µm. *p < 0.05 versus control group; ^#^ *p* < 0.05 versus PVR group; *n* = 3

To explore whether YAP inhibition could ameliorate PVR progression in vivo, the YAP inhibitor verteporfin was intraperitoneally administered to mice with PVR. Masson staining showed the presences of retinal fibrotic membranes (indicated by arrows) in the 8‐week PVR group, whereas reduced fibrotic epiretinal membrane formation (indicated by arrow) could be found in the verteporfin treatment group (Figure 4A). Verteporfin treatment partially attenuated the expression of the fibroblast markers CTGF, CYR61, ɑ‐SMA and ECM proteins (collagen I and fibronectin) (Figure 4B). To further explore whether verteporfin treatment affected retinal YAP expression in vivo, we examined YAP expression in the retinas of mice. The immunofluorescence staining revealed that treated with verteporfin could decrease the YAP accumulation in mice with PVR (Figure 4C).

### 3.5 YAP inhibition suppresses TGF‐β/Smad signalling

**FIGURE 5:**
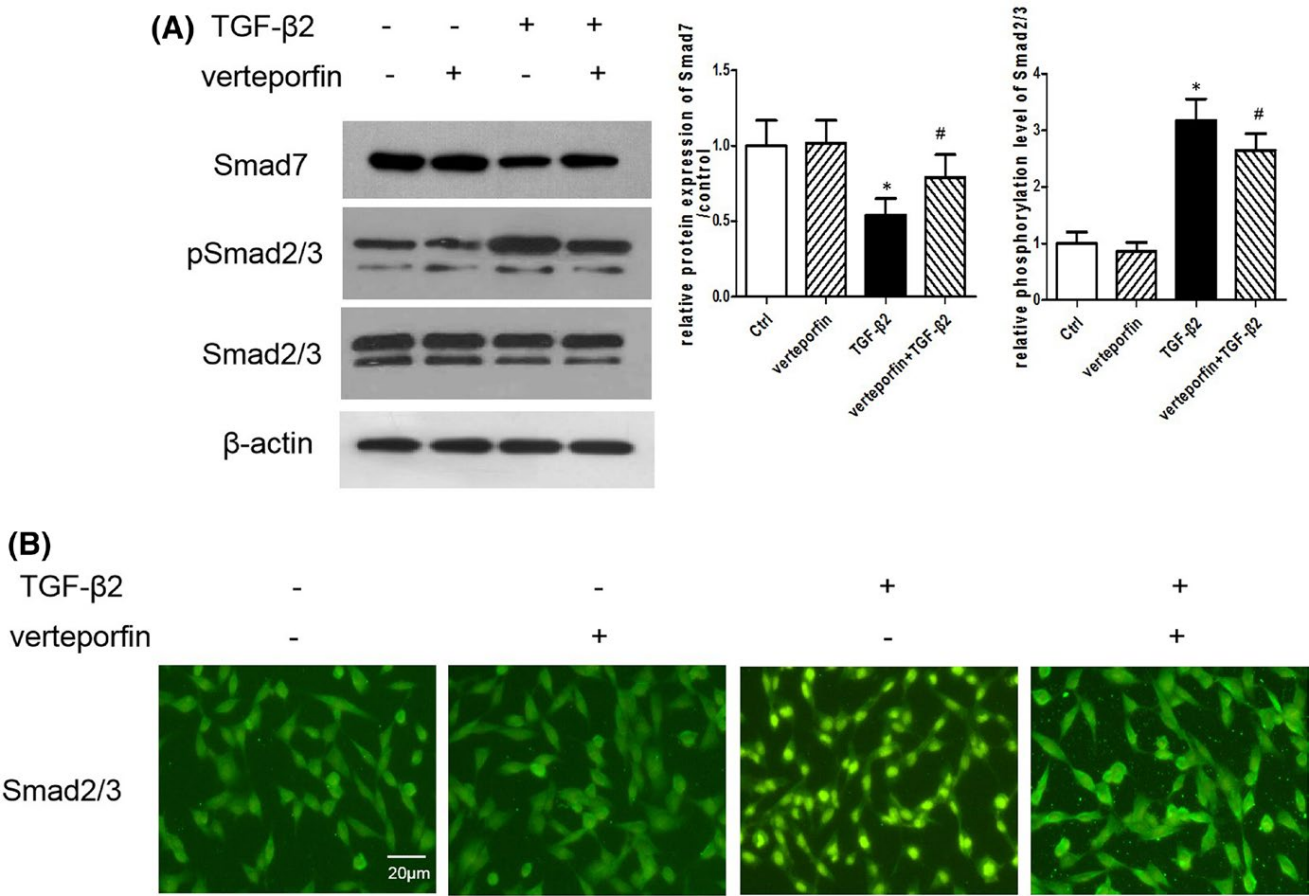
Verteporfin Suppresses TGF‐β/Smad Signaling. (A) Protein levels of Smad7, p‐Smad2, Smad2, p‐Smad3 and Smad3 in ARPE‐19 cells exposure to TGF‐β2 or/and verteporfin for 24 h were measured by Western blotting. (B) Expression of Smad2/3 in ARPE‐19 cells exposure to TGF‐β2 or/and verteporfin was detected by immunofluorescence staining. Scale bar = 20 µm. *p < 0.05 versus control group; ^#^ *p* < 0.05 versus TGF‐β2 group; *n* = 3

It has been shown that TGF‐β/Smad pathway is the principal pathway in EMT.^25^ Therefore, we analysed the expression of Smad proteins and phosphorylated Smad proteins in ARPE‐19 cells. Our results showed that the protein level of Smad7 (an inhibitory Smad protein) was downregulated in the TGF‐β2–treated group, while verteporfin reversed the TGF‐β2–induced downregulation of Smad7 (Figure 5A). The phosphorylation of Smad2/3 significantly increased in the TGF‐β2–treated group, and verteporfin decreased the TGF‐β2–induced phosphorylation of Smad2/3 (Figure 5A), suggesting that blockade treatment prevented Smad signalling activation. In addition, immunofluorescence examination showed that verteporfin inhibited the TGF‐β2–simulated translocation of Smad2/3 to the nucleus (Figure 5B).

**FIGURE 6:**
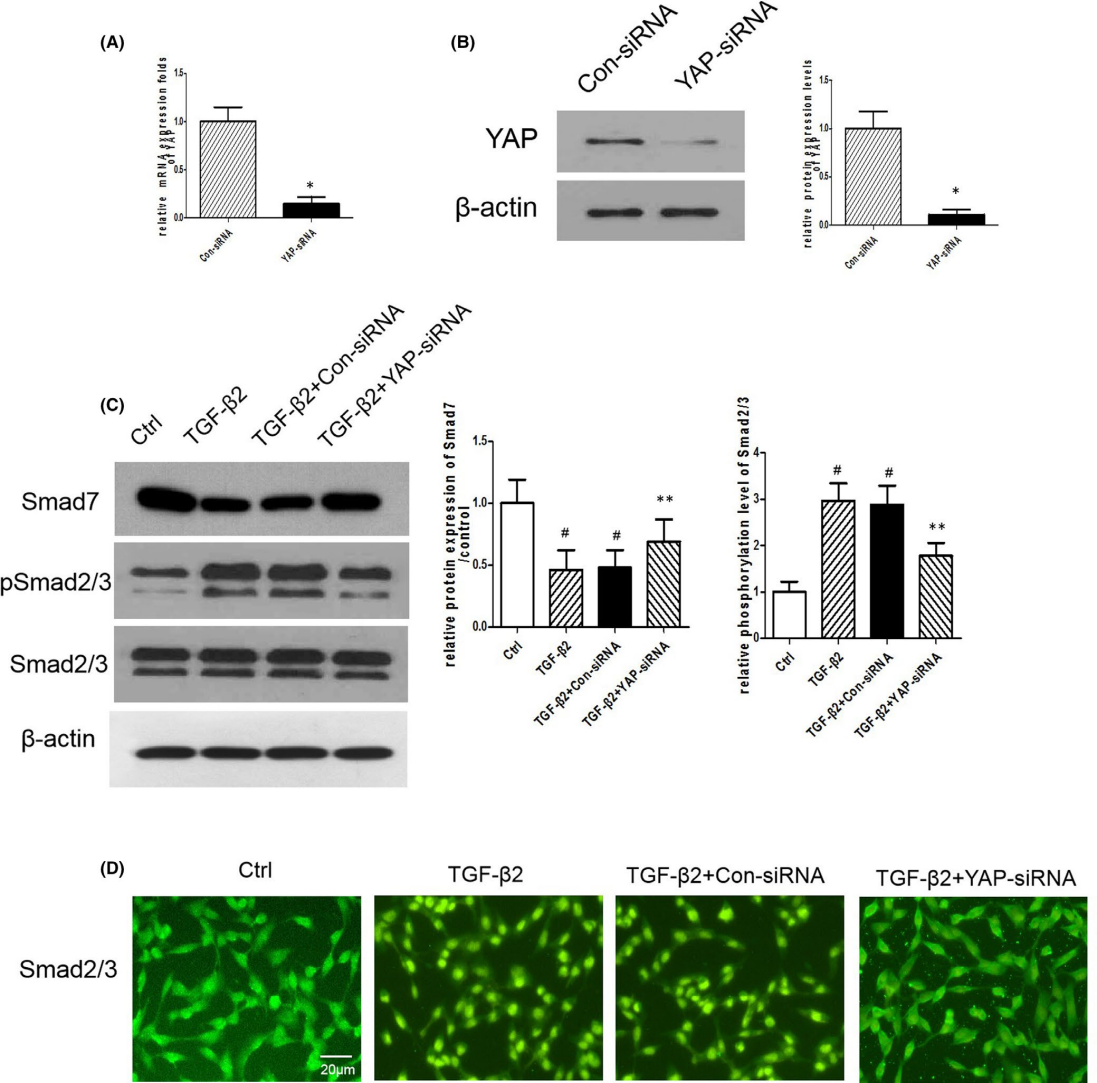
YAP‐siRNA Suppresses TGF‐β/Smad Signaling. (A, B) The expression of YAP in ARPE‐19 cells after transfection with YAP‐siRNA or Con‐siRNA was measured by RT‐PCR and Western blotting. *n* = 3. *p < 0.05 versus Con‐siRNA. (C) Protein levels of Smad7, p‐Smad2, Smad2, p‐Smad3 and Smad3 in ARPE‐19 cells transfected with YAP‐siRNA or Con‐siRNA and exposure to TGF‐β2 were measured by Western blotting. (D) Expression of Smad2/3 in ARPE‐19 cells transfected with YAP‐siRNA or Con‐siRNA and exposure to TGF‐β2 was detected by immunofluorescence staining. Scale bar = 20 µm. *p < 0.05 versus Con‐siRNA group; ^#^ *p* < 0.05 versus Control group; **p < 0.05 versus TGF‐β2 +Con‐siRNA group; *n* = 3

To further explore whether YAP was the crucial mediator of TGF‐β/Smad signalling, we designed YAP‐siRNA, and inhibitory efficiency of YAP‐siRNA was determined by RT‐PCR (Figure 6A) and Western blot (Figure 6B). Western blot analysis revealed that YAP‐siRNA could effectively increase the TGF‐β2–mediated downregulation of Smad7 and inhibit the TGF‐β2–mediated phosphorylation of Smad2/3 (Figure 6C). Immunofluorescence examination also showed that YAP knockdown effectively inhibited TGF‐β2–simulated Smad2/3 translocation to the nucleus (Figure 6D). Therefore, our results indicate that YAP is essential for the TGF‐β2–simulated Smad2/3 signalling pathway in ARPE‐19 cells.

## 4 DISCUSSION

In this study, we observed that YAP was upregulated in a PVR mouse model and during TGF‐β2–induced EMT in ARPE‐19 cells, suggesting that YAP may play a crucial role in the progression of PVR. Furthermore, our in vitro studies demonstrated that YAP knockdown maintained the RPE cell phenotype and alleviated TGF‐β2–induced activation and EMT, which are two important factors in PVR development. Moreover, the in vivo experiments showed that verteporfin attenuated PVR progression and fibrotic molecular changes in mouse model, which was complied with our in vitro study. Furthermore, YAP was essential for the TGF‐β2–mediated nuclear translocation and phosphorylation of Smad2/3. We also showed that YAP knockdown significantly inhibited the TGF‐β2–mediated fibrotic EMT process in ARPE‐19 cells. In summary, our research provides new ideas for the molecular mechanism of PVR.

Epithelial‐mesenchymal transition plays a key role in the development of PVR, but the exact mechanism by which EMT occurs in RPE cells is still unclear.^26^ Although a recent study revealed that glial cell migration is involved in the progression of PVR, RPE cells are thought to be a major factor in PVR‐induced EMT.^27^ In actual clinical situations, ophthalmologists usually observe floating pigment cells in the vitreous fluid of the eyes of RD patients.^28^ It has been shown that these floating pigment cells, presumably RPE cells, attach to the surface of the retina, undergo EMT and migrate as fibrotic cells.^29^ Miao et al.^30^ found that the loss of cell‐cell adhesion was the cause of EMT process and RPE cell proliferation. The epithelial markers, involving E‐cadherin and ZO‐1, have been proven to play an important role in maintaining cell proliferation and epithelial differentiation.^31^, ^32^ In our study, we found that knockdown of YAP inhibited the TGF‐β2–mediated increase in α‐SMA and fibronectin and decrease in ZO‐1 and E‐cadherin in ARPE‐19 cells. In addition, our study also showed that YAP knockdown decreased TGF‐β2–induced invasion, migration and gel contraction during the EMT process in vitro, which was complied with the research of Wang et al.^33^

In view of the crucial role of YAP in the response of cells to microenvironment stiffness, YAP has been shown to be associated with pathological fibrosis.^34^ In fact, the upregulation of YAP is observed in renal, liver and lung fibrosis.^35^, ^36^ It has been found that YAP is associated with TGF‐β‐Smad signalling during the fibrotic process.^37^ Consistent with these studies, we found that YAP was required for TGF‐β2–induced fibrosis in ARPE‐19 cells. Additionally, pharmacological and genetic suppression of YAP could abolish the ECM production and contraction induced by TGF‐β2. Importantly, our results showed that YAP could regulate the transcription of fibrotic genes (CTGF and CYR61) in ARPE‐19 cells. CTGF and CYR61 are proven to be downstream targets of YAP and act as signalling molecules that are relevant for the progression of pathogenic fibrosis.^15^ In our study, we showed that YAP knockdown ameliorated the TGF‐β2–induced increase in CTGF/CYR61 proteins in ARPE‐19 cells. In addition, the in vivo studies also showed that verteporfin attenuated the upregulation of CTGF/CYR61 proteins in the PVR mouse model. Verteporfin has been approved by the US Food and Drug Administration (FDA) for clinical use as a therapeutic approach for AMD.^38^ Our study indicates that strategies targeting YAP represent promising therapeutic options for preventing pathogenic fibrosis in PVR patients.

Proteins of the TGF‐β family are considered to be major regulators of pathogenic fibrosis, and TGF‐β2 is the main isoform of the TGF‐β protein family that is expressed in the eyes.^39^ In the canonical signalling pathway, TGF‐β mediates the phosphorylation of Smad2/3 and the subsequent transcription of fibrotic genes in several tissues, involving the kidney, lung and liver.^40^, ^41^, ^42^ It has been shown that increased expression of TGF‐β2 and CTGF was detected in PVR vitreous specimens.^27^ These cytokines may be included in the process of PVR. A previous study found an association between TGF‐β‐Smad signalling and YAP in the pathophysiology of TGF‐β2–mediated conjunctival fibrosis.^43^ Our study showed that YAP, as a major signalling component of the mechanical response, effectively regulated TGF‐β2–induced fibrosis by regulating Smad2/3 signalling. In our study, pharmacological and genetic inhibition of YAP reduced the phosphorylation and nuclear translocation of the Smad2/3 protein in ARPE‐19 cells, suggesting that the classic Smad signalling pathway can be inhibited by YAP knockdown treatment. In view of the results, YAP‐mediated fibrosis was closely related to TGF‐β2 signalling and contributed to PVR pathogenesis.

In summary, the present study revealed that YAP played a key role in TGF‐β2–induced retinal fibrosis during PVR progression. Our work provides the evidence for a role of YAP in inhibition of PVR and the potential use of YAP as a target in PVR therapeutic intervention. In addition, verteporfin, as a YAP inhibitor, has therapeutic potential to prevent retinal fibrosis after vitroretinal surgery. Future studies should further characterize the function of YAP in vivo.

## CONFLICT OF INTEREST

The authors state that they do not have any financial interest or other relationship with any product manufacturer or provider of services discussed in this article. The authors also do not discuss the use of off‐label products, which include unlabelled, unapproved, or investigative products or devices.

## AUTHOR CONTRIBUTIONS

**Wei Zhang:** Conceptualization (equal); Data curation (equal); Formal analysis (equal); Funding acquisition (equal); Investigation (equal); Methodology (equal); Project administration (equal); Validation (equal); Visualization (equal); Writing‐original draft (equal); Writing‐review & editing (equal). **Jing Li:** Conceptualization (equal); Investigation (equal); Methodology (equal); Software (equal); Supervision (equal); Validation (equal).

## Data Availability

The data will be available from the corresponding author by reasonable request.
